# *Cis*- and *trans*-regulation in X inactivation

**DOI:** 10.1007/s00412-015-0525-x

**Published:** 2015-07-22

**Authors:** Joke G. van Bemmel, Hegias Mira-Bontenbal, Joost Gribnau

**Affiliations:** Mammalian Developmental Epigenetics Group, Institute Curie, CNRS UMR3215, INSERM U934, Paris, France; Department of Developmental Biology, Erasmus MC, Wytemaweg 80, 3015 CN Rotterdam, The Netherlands

## Abstract

Female mammalian cells compensate dosage of X-linked gene expression through the inactivation of one of their two X chromosomes. X chromosome inactivation (XCI) in eutherians is dependent on the non-coding RNA *Xist* that is up-regulated from the future inactive X chromosome, coating it and recruiting factors involved in silencing and altering its chromatin state. *Xist* lies within the X-inactivation center (Xic), a region on the X that is required for XCI, and is regulated in *cis* by elements on the X chromosome and *in trans* by diffusible factors. In this review, we summarize the latest results in *cis*- and *trans*-regulation of the Xic. We discuss how the organization of the *Xic* in topologically associating domains is important for XCI (*cis*-regulation) and how proteins in the pluripotent state and upon development or differentiation of embryonic stem cells control proper inactivation of one X chromosome (*trans*-regulation).

## Introduction

X chromosome inactivation (XCI) is the mechanism by which female mammalian cells achieve dosage compensation of X-linked gene expression. Throughout eutherian evolution, our sex chromosomes adopted distinct fates; the X chromosome has maintained most of the original genes, whereas the Y chromosome has degenerated in a chromosome with low levels of genetic diversity, many repetitive sequences and most of the genes still present are involved in male fertility (Graves 2006). Degeneration of the Y chromosome provided a potential imbalance in X-chromosomal versus autosomal gene products. Susumu Ohno therefore predicted a twofold transcriptional up-regulation of X-linked genes (Ohno 1967), a hypothesis that was initially confirmed (Nguyen and Disteche 2006), but later contested (Lin et al. 2012; Chen and Zhang 2015). These findings indicate that dosage compensation is limited to subsets of genes, being more pronounced for highly expressed genes and genes encoding proteins acting in complexes (Deng et al. 2011). An obvious consequence of this up-regulation is that female mammalian cells would express X-linked genes at twice the level compared to autosomal genes. To compensate for these potential dosage differences, female cells inactivate one of the two X chromosomes. This XCI process occurs early in murine development in two waves. Imprinted XCI (iXCI) is initiated early during pre-implantation development in all cells of the embryo, and leads to exclusive inactivation of the paternally inherited X chromosome (Xp). In the inner cell mass (ICM) of the pre-implantation embryo, the inactive Xp is reactivated (Mak et al. 2004; Okamoto et al. 2004) followed by a second wave of XCI in the epiblast, around E5.5 of development. This second wave is random with respect to the parental origin of the X chromosome, resulting in XCI of either the maternal or paternal X chromosome. The silent state of the inactivated chromosome is mitotically inherited and maintained in the daughter cells.

Extensive work over several decades uncovered a master regulatory region critical for XCI, the X inactivation center (Xic). The Xic contains protein-coding genes, non-coding genes and their *cis*-regulatory elements that ensure the proper initiation and exclusive inactivation of one X chromosome in every female cell. The main actor of XCI is *Xist*, which is a non-coding RNA gene, located within the Xic. *Xist* is up-regulated at the onset of XCI, coating the future inactive X chromosome. One of the earliest detectable events following *Xist* spreading is the depletion of RNA Pol II and transcription factors from the coated chromosome (Chaumeil et al. 2006). Afterwards, active histone modifications are lost from the inactive X (Chaumeil et al. 2002). Subsequently, the Polycomb-recruiting complexes 1 and 2 (PRC1 and 2) are recruited to the inactive X, marking histones with repressive modifications, such as H3K27me3 and H2AK119Ub (de Napoles et al. 2004; Fang et al. 2004; Plath et al. 2004). One of the final steps in the silencing of the X is the establishment of repressive CpG methylation at promoters and CpG islands (Lock et al. 1987; Gendrel et al. 2013).

Female ICM-derived embryonic stem cells (ESCs) contain two active X chromosomes (Xa's), and their differentiation in vitro results in initiation of random XCI (rXCI), thereby providing a powerful model system to study XCI. Prior to XCI initiation, *Xist* expression is kept at low levels by means of different genetic elements and transcription factors. The best-established negative regulator of *Xist* is *Tsix*. Similar to *Xist*, *Tsix* is also a non-coding gene, overlaps with, and is transcribed antisense to *Xist*. The exact mechanism by which *Tsix* represses *Xist* is however unclear, but RNA mediated recruitment of chromatin remodelers to the *Xist* promoter, and transcriptional interference mechanisms have been implicated (Stavropoulos et al. 2001; Luikenhuis et al. 2001; Shibata and Lee 2004; Sado et al. 2006). The factors that regulate *Xist* and *Tsix* transcription, and thus XCI, can be classified in two categories, a *cis*-regulatory network and a *trans*-regulatory network. The *cis*-regulatory network is embedded within the X chromosome representing the classical Xic, and is composed mainly and possibly exclusively of genetic factors, that act in *cis* by DNA interactions. On the contrary, *trans*-acting regulatory factors are diffusible, and thus can act from a distance and can be autosomally encoded or X-linked.

Recent exciting work has shed new light on the complex interplay of the different *cis*- and *trans*-acting factors in the regulation of rXCI in mouse. In this review, we describe these latest findings with respect to the regulation of rXCI, which involve all levels of gene regulation, including *cis*-regulatory elements, transcription factor networks, chromatin modifications, and higher-order chromatin structure.

## The *cis*-regulatory environment and its spatial separation

The key *cis*-acting regulatory elements in XCI are located within the Xic, which has been delineated by genetic studies in cell lines harboring X chromosomal deletions and X to autosome translocations (Rastan 1983; Rastan and Robertson 1985; Heard et al. 1994; Lee et al. 1996). These studies revealed a ~500-kb region on the X to be required for the initiation of XCI. Close examination of this region revealed *Xist*, but also the presence of several other non-coding RNA genes in its close vicinity, many of which are involved in the positive or negative regulation of XCI. For most of them, it remains to be determined if they regulate *Xist* or *Tsix* expression at the RNA level via their transcript, or at the DNA level via the regulatory elements contained within them.

**Fig. 1:**
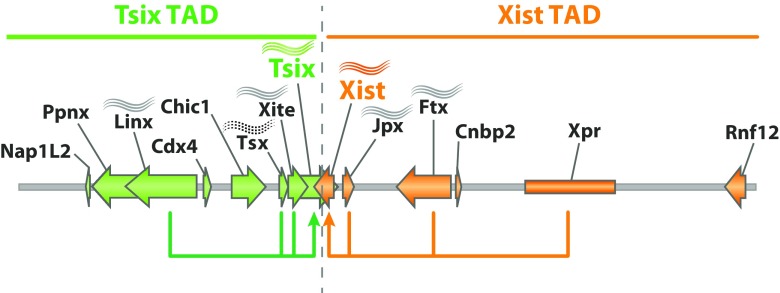
The *cis*-regulatory environment and its spatial separation. The Xic is divided (light gray dashed line) in two topologically associated domains (TADs). *Xist* and *Tsix* reside in distinct TADs each harboring their own (putative) regulatory elements: the *Xist* TAD includes *Jpx*, *Ftx*, *Xpr*, and *Rnf12*, while the *Tsix* TAD includes *Linx*, *Chic1*, *Tsx*, and *Xite*. Genes within each TAD are regulated coordinately during differentiation. Wave symbols on top of genes indicate non-coding genes. *Tsx* appears to have functions as a coding and non-coding gene

Two of these non-coding RNA genes, *Jpx* and *Ftx*, are located upstream of *Xist* (Fig. 1). Both genes escape XCI and are therefore co-expressed with *Xist* during differentiation (Tian et al. 2010; Chureau et al. 2011). *Ftx* deletion in male ES cells caused decreased expression of *Xist*, *Jpx*, and *Tsix*, but since this was only analyzed in male cells, *cis* or *trans* effects could not be distinguished in this study (Chureau et al. 2011). A heterozygous deletion of *Jpx* in female ES cells has been reported to decrease *Xist* up-regulation in both alleles, which could be rescued by an autosomal transgene, suggesting *Jpx* to act in *trans* (Tian et al. 2010). The reported *trans*-activity of *Jpx* has been proposed to occur by dose-dependent eviction of CTCF at the *Xist* promoter (Sun et al. 2013). However, other previous, as well as recent, *Jpx-* and *Ft*x-containing transgene studies did not observe any ectopic *Xist* up-regulation (Heard et al. 1999; Jonkers et al. 2009; Barakat et al. 2014), and a heterozygous deletion of a *Jpx-* and *Ftx*-containing region in female mESCs did not affect *Xist* up-regulation from the wild-type X chromosome (Barakat et al. 2014). These results suggest that *Jpx* and *Ftx* activate *Xist* mainly through *cis*-mediated mechanisms.

Just like the *cis*-regulators of *Xist* are located upstream of *Xist*, the positive regulators of *Tsix* all reside in a region upstream of the *Tsix* promoter. These include the RNA *Tsx* and the enhancer *Xite* (Simmler et al. 1996; Ogawa and Lee 2003; Anguera et al. 2011), as well as the more recently identified putative regulatory elements contained in *Linx* and *Chic1* (Nora et al. 2012). Deletions of *Xite* and *Tsx* both result in mild effects in XCI and expression of *Xist. Xite*, which is located just upstream of *Tsix*, acts in *cis* as an enhancer of *Tsix*, but is itself also transcribed (Ogawa and Lee 2003). Interestingly, the putative regulatory elements within *Linx* and *Chic1* were identified due to their long range *cis*-interactions with the *Tsix* promoter or its enhancer *Xite* (Nora et al. 2012). In addition to containing putative regulatory elements, the *Linx* gene also gives rise to a long non-coding RNA. Furthermore, *Tsix* transcription is found to be regulated by the transcribed DXPas34-repeat region, which is located 750 bp downstream of the *Tsix* promoter (Debrand et al. 1999; Stavropoulos et al. 2005; Vigneau et al. 2006; Cohen et al. 2007).

The above described localization of the *cis*-regulatory elements with respect to the *Xist* and *Tsix* promoters, shows a partitioning of the *cis*-regulatory environment in two regions (Fig. 1), one covering the regulatory elements of *Tsix*, the other those of *Xist*. Thanks to 5C (3C-based analysis of many selected loci in parallel), which maps the frequency/propensity of chromatin interactions, the Xic was found to be separated not only functionally but also spatially. The chromosome interaction map of the Xic displays a structural organization in two spatially separated domains, so called topologically associating domains (TADs) (Nora et al. 2012). Interestingly, the border of these two TADs is located exactly in between the promoters of *Xist* and *Tsix*, thereby segregating the *Xist* promoter and its activators from the *Tsix* promoter and its respective regulators.

Does this spatial segregation ensure functional separation and oppositely regulated transcription, or is the spatial organization merely a consequence of the transcriptional status of *Xist* and its antisense regulator *Tsix*? If it is causal, then what determines this organization? What delimits TADs? Is TAD border formation driven by specific features or is it rather the consequence of interactions being favored elsewhere? Several findings indicate TAD organization to be important for proper transcriptional regulation. First of all, using HiC, it became clear that the entire mouse genome as well as the human and the Drosophila genomes are organized in TADs, and that”boundaries” between them seem to be conserved between species (Sexton et al. 2012; Dixon et al. 2012; Hou et al. 2012), suggesting this organization to be functionally relevant. Second, several studies indicate that TADs can be considered as discrete units of gene regulation since genes within the same TAD tend to be transcriptionally regulated in a coordinated fashion (Nora et al. 2012; Le Dily et al. 2014) and the majority of promoter–enhancer pairs reside within TADs (Kleinjan and Coutinho 2009; Shen et al. 2012; Smallwood and Ren 2013; Nora et al. 2013; Symmons et al. 2014). Third, a 58-kb deletion of the *Xist* and *Tsix* TAD border region results in decreased spatial separation of the two domains and illegitimate interactions between sequences within the domains, which co-occurred with altered gene expression of the genes in these domains (Monkhorst et al. 2008; Nora et al. 2012).

The mechanisms behind TAD formation and the boundaries between them remain to be determined however. The genome-wide analyses by Dixon et al. showed TAD borders to be enriched for several genomic elements, suggesting a role in TAD boundary formation (Dixon et al. 2012). Especially the architectural proteins CTCF, cohesin and mediator are considered as favorite candidates to be causal in establishing topological domain structure. Distinct combinations of these architectural proteins are often but not always found at TAD boundaries, long range interacting loci, and short range intraTAD interacting loci (Li et al. 2013; Phillips-Cremins et al. 2013; Sofueva et al. 2013; Rao et al. 2014). Cleavage or depletion of the cohesin complex or CTCF was found to decrease intradomain interactions and to increase interdomain interactions but notably did not lead to a total loss of TADs (Seitan et al. 2013; Sofueva et al. 2013; Zuin et al. 2014). These results indicate that architectural proteins could contribute to TAD formation by executing an insulator function at the border and/or by mediating intra-TAD contacts, which by themselves could help to prevent inter-TAD contacts and consequently contribute to shaping the boundary.

Interestingly, the TAD border that separates the *Xist* and *Tsix* regulatory environments is bound by CTCF, but not by cohesin. A 2.3-kb deletion of the CTCF site-containing region in female mES cells resulted in improper transcriptional regulation of *Xist* and *Tsix* during differentiation (Spencer et al. 2011), suggesting that this CTCF binding site is indeed involved in the spatial separation of the regulatory elements of *Xist* and *Tsix*, even if not bound by cohesin. However, the effect of this deletion on chromatin interactions was not monitored, so it remains to be determined if this effect is really due to reduced spatial separation. In addition CTCF, often together with cohesin, is frequently bound inside the *Xist* and *Tsix* TADs. The binding sites overlap with the (putative) *cis*-regulatory elements and promoters of *Xist* and *Tsix*, which are all intra-TAD interacting loci. As mentioned above, these intra-TAD CTCF- and cohesin-bound loci could also contribute to the sharpness of the boundary between the *Xist* and *Tsix* TADs (Giorgetti et al. 2014).

5C and Hi-C contact maps can be considered to represent the contact frequency or probability across a population, and TADs represent an average chromatin conformation within many cells. The use of FISH probes covering entire TADs and super resolution imaging showed their size and their degree of co-localization to differ from one cell to another (Nora et al. 2012). Also predictive polymer modeling of TAD conformation represented by the population data has been used to predict an ensemble of conformations, revealing the chromatin conformation within the *Tsix* TAD to be highly variable between cells (Giorgetti et al. 2014). In this model, for instance, the *Tsix* promoter interacts with one or more of its (putative) regulatory elements, *Xite*, *Chic1* and *Linx* in only a certain percentage of cells. Transcriptional activity of a locus is known to vary from cell to cell, and by combining the model’s predictions with high-resolution DNA FISH and quantitative RNA FISH, a relationship was confirmed between the transcriptional activity and the chromatin conformation of the *Tsix* alleles at single cell level. Notably, conformation and transcriptional activity were found to vary between the two *Tsix* alleles within the same cell. Such fluctuations could be responsible for asymmetric *Tsix* transcription and thus asymmetric *Xist* up-regulation (Giorgetti et al. 2014).

## *Trans*-regulation of XCI

**Fig. 2:**
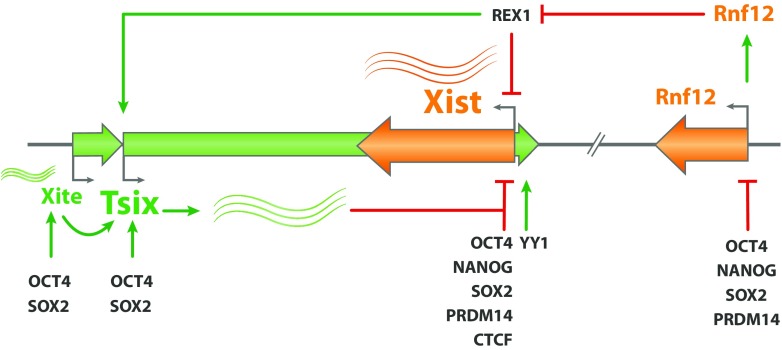
Trans-regulation of the Xic in ES cells is affected by different diffusible factors. The autosomally encoded pluripotency factor network directly represses activation on *Xist* is ES cells, or through activation of the repressor of *Xist*, *Tsix*. OCT4, SOX2, NANOG, and PRDM14 also repress the activator of XCI, Rnf12. Activation of XCI involves the dose-dependent break down of REX1 by RNF12. REX1 represses *Xist* and activates *Tsix*. Autosomally encoded YY1 activates *Xist* transcription by competing with REX1 for binding to *Xist* regulatory elements

*Trans*-regulation of the Xic refers to the regulation of genes within the Xic by diffusible factors and can be inhibitory or activating. Years of research in the XCI field have uncovered a crucial role for several pluripotency factors in the regulation of XCI, thereby providing an important link between loss of pluripotency and XCI initiation. Several studies have implicated OCT2, NANOG, SOX2, REX1 and PRDM14 to act as inhibitors of XCI either directly, by repressing *Xist* or activating *Tsix*, or indirectly by repressing activators of XCI (Fig. 2) (Navarro et al. 2008; Donohoe et al. 2009; Ma et al. 2011; Navarro et al. 2011; Gontan et al. 2012; Payer et al. 2013). Removal of OCT4 from male ES cells results in differentiation and up-regulation of *Xist* to similar levels as in differentiating female cells (Navarro et al. 2008), while OCT4 removal from differentiating female ES cells results in biallelic up-regulation of *Xist* (Donohoe et al. 2009). OCT4, NANOG, SOX2, and PRDM14 strongly bind to *Xist* intron 1, which led to the hypothesis that their repressive action on *Xist* is mediated through this region. However, knockout studies deleting the *Xist* intron 1 region indicated that it is dispensable for *Xist* repression (Barakat et al. 2011; Minkovsky et al. 2013). Nevertheless, deletion of the *Xist* intron 1 binding site together with the *Tsix* positive regulatory region, DXPas34, that is also regulated by OCT4, results in de-repression of *Xist*, not found with the individual mutations, suggesting that multiple redundant mechanisms are in place to repress *Xist* (Donohoe et al. 2009; Nesterova et al. 2011). REX1, another pluripotency factor, also acts as an inhibitor of XCI by binding to the DXPas34 repetitive sequence associated to the *Tsix* promoter, allowing proper transcription elongation of the *Tsix* transcript which in turns represses *Xist* (Navarro et al. 2010). In addition, REX1 was later demonstrated to directly repress *Xist* by binding to the *Xist* promoter and the promoter downstream region (Gontan et al. 2012). REX1-mediated repression of *Xist* probably involves competition of REX1 with its paralog and *Xist* activator YY1 for binding to the same sites (Makhlouf et al. 2014). REX1’s role is therefore twofold: it acts at the level of *Xist* repression as well as the level of *Tsix* activation to inhibit XCI in ESCs. YY1, in addition to its role as a direct *Xist* activator, binds to the nucleation center located within 1Kbp from *Xist* repeat A, facilitating docking *Xist* RNA molecules on the Xi (Jeon and Lee 2011).

OCT4, NANOG, SOX2, and PRDM14 have also been reported to indirectly repress XCI by inhibiting *Rnf12* expression (Fig. 2) (Navarro et al. 2011). *Rnf12* is an XCI activator encoding an E3-ubiquitin ligase involved in the dose-dependent degradation of REX1 (Navarro et al. 2011; Gontan et al. 2012; Payer et al. 2013). OCT4, NANOG, SOX2, and PRDM14 bind near the *Rnf12* promoter, and depletion of NANOG or OCT4 results in increased *Rnf12* expression (Navarro et al. 2011). PRDM14 is a transcriptional regulator that is specifically expressed in ESCs and primordial germ cells where it has been implicated in epigenetic reprogramming (Yamaji et al. 2008, 2013). Depletion of PRDM14 also results in *Xist* up-regulation, which might involve loss of PRDM14 binding to the intron 1 region (Ma et al. 2011; Payer et al. 2013). PRDM14 may also act indirectly by repressing *Rnf12* expression, as *Prdm14*^−/−^ ESCs show decreased binding of PRC2 to and reduced deposition of the H3K27me3 repressive mark on the *Rnf12* promoter region, which correlates with a four-fold increase in *Rnf12* expression. These findings indicate that several mechanisms, many of them closely linked to the pluripotency factor network, ensure proper repression of *Xist* in mouse ESCs and the timed and proper initiation of XCI upon ESC differentiation.

*Rnf12* is located 500 Kb upstream of *Xist. Rnf12* expression is low in mESCs but up-regulated upon differentiation, aided by decreasing levels of the pluripotent factors that repress *Rnf12* expression in the pluripotent state. RNF12 over-expression results in inactivation of the single X chromosome and of both X chromosomes in male and female differentiating ESCs, respectively (Jonkers et al. 2009). *Rnf12*^+/−^ ESCs manage to inactivate one X chromosome upon differentiation, albeit at a reduced rate compared to wild-type ESCs (Barakat et al. 2011). Since *Rnf12*^+/−^ cells are technically like male cells in terms of RNF12 dosage, this result suggests the existence of additional factors that activate XCI in female differentiating ESCs. Nevertheless, *Rnf12*^−/−^ ES cells completely fail to up-regulate *Xist* during in vitro differentiation, suggesting that RNF12 is essential for XCI in vitro (Barakat et al. 2011). *Rnf12* expression (from the Xa) is even continuously required to establish the Xi, providing a strong feedback mechanism ensuring XCI of one X chromosome only (Barakat et al. 2014). Effects of complete loss of RNF12 in ESCs have been reported to be less severe in a different study (Shin et al. 2010). These phenotypic differences might be explained by the different knockout constructions with both of them resulting in a truncated version of RNF12 or by the distinct genetic backgrounds of the ESCs studied, as the expression level of several pluripotency factors acting as XCI-inhibitors varies between ESCs obtained from different mouse crosses (Sharova et al. 2007). In mice, *Rnf12*^−/+^ females with a maternally transmitted null allele die in utero, contrarily to *Rnf12*^+/−^ female mice with a paternally transmitted mutant allele (Shin et al. 2010). Further investigation highlighted the absence of *Xist* clouds and loss of inactivation of the paternal X chromosome during iXCI in *Rnf12*^−/+^ embryos, leading to abnormal extraembryonic tissue development and eventually death of the embryo. This phenotype was attributed to the loss of the maternal pool of RNF12 after a conditional knockout of *Rnf12* in the developing oocyte and the requirement for high levels of RNF12 to initiate iXCI.

In a more recent study, the role of RNF12 in rXCI was addressed in vivo (Shin et al. 2014). Using conditional *Rnf12* knockout alleles deleting *Rnf12* exclusively in the ICM by means of a Cre recombinase under the control of the *Sox2* promoter, in this way bypassing RNF12’s role in iXCI in extraembryonic tissues, full knockout *Rnf12* females were born at normal Mendelian ratios. RNA-FISH analysis on tissues of adult *Rnf12* knockout females shows normal XCI of a single X chromosome, suggesting that RNF12 is dispensable for rXCI in vivo (Shin et al. 2014). Selection against cells that failed to initiate XCI might have occurred. Nevertheless, these findings also suggest that XCI might be more robust in vivo than in vitro, and clearly, more studies are required to establish the role of the genetic background. Altogether, *trans*-regulation of the Xic by the pluripotency factor network and low levels of RNF12 limit the up-regulation of *Xist* in ESCs, maintaining the two X chromosomes in an active state.

The pluripotency network is tightly linked to the presence of two active X chromosomes in female cells. The reverse, that is, two Xa’s stabilizing the pluripotent state, has been suggested in a recent report by Schulz and colleagues (Schulz et al. 2014). Genome-wide transcriptome analysis of XX, XO, and XY ESCs indicated that double dosage of the X chromosomal gene products delays differentiation of XX ESCs compared to X0 or XY ESCs, stabilizing the pluripotent state. The presence of two Xa’s inhibits the Mek and Gsk3 pathways in ESCs, similarly to 2i-containing growth medium, and reduces the levels of de novo methyltransferases 3a and 3b (DNMT3a/3b), which correlates with previous reports showing global DNA hypomethylation of female ESCs (Zvetkova et al. 2005; Habibi et al. 2013). The ectopic induction of *Xist* in XX ESCs and concomitant XCI led to increased DNMT3a/3b expression resulting in similar DNA methylation levels in XX and XO control ESCs. Based on these observations, the authors suggest that the presence of two Xa’s inhibits exit from the naïve pluripotent state and differentiation (Schulz et al. 2014). Mechanistically, which genes on the two active X chromosomes stabilize the pluripotent state remains an open question.

## The Xic at work

Several mechanisms have been proposed to explain exclusive inactivation of a single X. Studies with heterokaryons obtained through fusion of male and female cells indicate that XCI is equally well initiated in the male and female nuclei indicating that all the regulatory cues involved in initiation of XCI are diffusible and cross the nuclear membrane (Barakat et al. 2014). This argues against a role for pairing, of the Xic or the X pairing regulatory regions, in the initiation of XCI (Xu et al. 2006, 2007; Bacher et al. 2006; Augui et al. 2007). XCI inhibitors including a wide range of pluripotency factors set the threshold for XCI activation (Barakat et al. 2010). Activators of XCI, including RNF12, are X-encoded and will thus be expressed at a twofold higher level in female cells compared to male cells. The higher level of X-encoded activators in female cells will overcome the threshold set by the autosomally encoded inhibitors, thereby ensuring female exclusive initiation of XCI. *Xist* is also activated by autosomally encoded factors, including YY1, which competes with REX1 for binding to the *Xist* regulatory region located downstream of the *Xist* promoter (Makhlouf et al. 2014; Chapman et al. 2014). These autosomally encoded *Xist* activators, however, will be expressed at equal levels in female and male cells, and are therefore not to be considered as activators of XCI. During development or upon ESC differentiation, the drop in expression of pluripotency factors and the concomitant increase in the expression of XCI activators lead to *Xist* up-regulation. Initiation of XCI in female cells is most likely a stochastic process and might happen on any of the two X chromosomes in a given time span (Monkhorst et al. 2009). Exclusive initiation of XCI on one X chromosome might be facilitated by inherent differences in transcriptional activity and higher-order chromatin structure. Since fluctuations in internal TAD conformation are related to variability in transcriptional activity, stochastic interactions between the *Tsix* promoter and its regulatory sequences could facilitate asymmetric *Tsix* activity between alleles, thereby causing up-regulation of *Xist* from one allele and not from the other (Giorgetti et al. 2014). This does not prevent the two alleles in the same cell from adopting the same fate, and therefore fast feedback mechanisms must exist. This includes the rapid turnover of RNF12 and REX1, and the continuous requirement for one active copy of *Rnf12*, preventing XCI on all except one X chromosome. Indeed, the half-life of REX1 was determined to be in the order of several minutes, and also RNF12 is very unstable through auto-ubiquitination (Gontan et al. 2012). In addition, the close proximity of *Rnf12* and *Xist* likely facilitates feedback through rapid silencing of *Rnf12* upon *Xist* up-regulation in iXCI and rXCI (Patrat et al. 2009; Barakat et al. 2014). Finally, the close link between the presence of two Xa’s, expression of pluripotency factors, and repression of XCI puts a brake on differentiation of cells that have not yet initiated XCI. Together, all these regulatory mechanisms guarantee a robust and highly efficient XCI process.

In several mouse strains and in human, rXCI is skewed towards inactivation of the Xp or Xm (Cattanach and Williams 1972; Gale et al. 1992). Variation in regulatory elements resulting in allelic differences in transcriptional activity of *Xist* and *Tsix* could potentially offer an explanation for skewing of XCI. Small genetic differences (i.e., SNPs) might also impact on the chromatin conformation, thereby causing allelic transcriptional biases explaining skewed X-inactivation. In such a case, these SNP-induced structural variations would represent the X controlling element (Xce), which has been genetically linked to skewing and is proposed to be located within a 1.8 mb region 3’ of the *Xist* promoter (Chadwick et al. 2006; Thorvaldsen et al. 2012). Allele-specific chromatin conformation capture studies or DNA-FISH-based compaction analysis in hybrid cells would be needed to test this hypothesis.

## Silencing and reactivation

The mechanisms underlying silencing of the X have been under intense scrutiny. *Xist* accumulation is followed by RNA Pol II and transcription factor exclusion (Chaumeil et al. 2006) and active histone mark removal (Chaumeil et al. 2002). Subsequently, PRC2 is recruited to the X that is silenced. PRC2-dependent H3K37me3 then signals PRC1 to monoubiquitylate histone H2AK119, although this order of events has been contested (Tavares et al. 2012). *Xist* and PRC2 take advantage of the three-dimensional structure of the chromosome to firstly silence active gene-rich regions that are in close proximity to the *Xist* locus in 3D, subsequently pulling gene-poor regions into the silencing compartment (Engreitz et al. 2013; Simon et al. 2013). Using probes to pull down *Xist* followed by mass-spectrometry (RAP-MS and ChIRP-MS), two independent studies identified proteins that interact with *Xist* and are necessary for its localization and/or silencing capacity (Chu et al. 2015; McHugh et al. 2015). One study revealed *Xist* to interact with SHARP, which recruits the SMRT co-repressor, activating HDAC3 implicated in deacetylation of histones on the Xi and chromatin compaction (McHugh et al. 2015). Knockdown studies indicated that SMRT and HDAC3 are required for *Xist*-dependent PRC2 recruitment to the Xi. A second study describes a different set of *Xist*-interacting proteins, of which HnrnpK and Spen specifically interact with *Xist* and are essential for silencing, but not localization to the Xi (Chu et al. 2015). Certain *Xist* interactors, such as Rmb15, Myef2, Hnrnpc, etc., are found in both studies, although other *Xist* interactors, such as PRC2, ATRX, CTCF, and YY1, previously identified by protein pool down followed by RNA-seq (CLIP-seq) were not identified in the *Xist*-specific pool down experiments (Zhao et al. 2008; Jeon and Lee 2011; Sarma et al. 2014; Kung et al. 2015). This discrepancy between *Xist*-mediated pool down of interacting proteins versus pool down of candidate proteins followed by RNA-seq could be explained by the different methods and systems used (male vs female cells) but could also hint at a role for many different factors providing redundancy to the system. This might involve other non-coding genes, including *Firre* which is X-linked but escapes XCI producing an RNA that, similar to *Xist*, is required for maintenance of H3K27me3 at the Xi, and nucleolar localization of the Xi (Yang et al. 2015).

These studies highlight the versatile mechanisms and robustness of the process involving non-coding RNAs and chromatin-modifying enzymes catalyzing histone modifications and CpG island methylation. XCI has therefore long been considered irreversible from the moment the Xi is established (Wutz and Jaenisch 2000), supported by studies with fibroblasts and neural progenitor cells revealing no robust reactivation after conditional knockout of *Xist* from the Xi, indicating that silencing is faithfully maintained through all daughter generations (Csankovszki et al. 2001; Splinter et al. 2011). This view was recently challenged by studies of Yildirim and colleagues, who deleted *Xist* in the blood compartment, which resulted in increased X-linked expression (Yildirim et al. 2013). Although allelic origin was not investigated, the authors suggested that *Xist* does play a role in maintenance of the inactive state in differentiated cells, at least in the blood compartment. Importantly, female mice develop myelofibrosis, leukemia, and other symptoms of the myeloproliferative neoplasm and myelodysplastic syndrome. The authors argue that reactivation of the X chromosome leads to genome-wide expression changes and deregulation of the cell cycle, DNA replication, and hematopoietic pathways, among other genetic pathways. It thus seems that the blood lineage is plastic for the reactivation but also inactivation of the X (Agrelo et al. 2009; Yildirim et al. 2013). Recently, reactivation of the X chromosome of female mouse embryonic fibroblasts has been shown to have no clear effect on global X-linked gene expression (Bhatnagar et al. 2014). Using an RNAi interference screen during differentiation of female mouse ESCs and in differentiated cells, the authors identified 13 *trans*-acting XCI factors (XCIFs) that are required for proper expression of *Xist* and/or localization of *Xist* to the Xi. These XCIFs include proteins involved in cell signaling, transcription, and ubiquitination, such as AURKA, SOX5, and RNF165, respectively. A mouse knockout model of one of the XCIFs, STC1, a poorly studied gene encoding a nuclear and cytoplasmic glycoprotein, shows the expected XCI phenotype, with most of *Stc1*^−/−^-derived MEFs displaying bi-allelic expression of four X-linked genes tested. More importantly, the presence of two Xa’s in differentiated cells of *Stc1*^−/−^ mice does not lead to over-expression of X-linked genes by RNA-seq analysis and qRT-PCR confirmation, which would explain why *Stc1*^−/−^ mice are fertile and phenotypically normal. Although the allelic origin of X-linked expression was not analyzed in this study, the increase of X-linked gene expression expected from the reactivation of the Xi might be absent due to cell selection or dampened by hitherto unknown compensatory mechanisms, similar to compensatory mechanisms described for Down syndrome (Aït Yahya-Graison et al. 2007).

## Conclusions and perspectives

XCI is regulated at different levels simultaneously. Non-coding RNAs and *cis*-regulatory elements on the X chromosome are critical to maintain proper repression of *Xist* in the pluripotent state. On the other hand, proteins either encoded by the X, such as RNF12, or encoded by autosomes, such as the pluripotent transcription factor family, act at a distance, in *trans*. Despite the progress made, several open questions remain in the field. Are the contrasting results obtained with independently generated deletions of *Rnf12* the consequence of differences in the genetic background or related to fragments of the gene that where left intact? How many XCI *trans*-activators regulate rXCI? In addition, the mechanism by which *Tsix* and the other ncRNAs within the Xic regulate *Xist* is still being debated. Is transcription per se, i.e., activity, enough to repress or activate *Xist*, are the non-coding transcripts involved, or rather the regulatory elements contained within these genes and is the higher-order chromatin structure instructive or consequence in the regulation of XCI? Finally, what is the role of all the novel and previously identified *Xist* interactors in establishment and maintenance of the Xi, and how do these findings observed in mouse relate to human? These and many other intriguing questions are awaiting to be addressed soon.
